# Land use history and population dynamics of free-standing figs in a maturing forest

**DOI:** 10.1371/journal.pone.0177060

**Published:** 2017-05-24

**Authors:** Larissa Albrecht, Robert F. Stallard, Elisabeth K. V. Kalko

**Affiliations:** 1 Institute of Experimental Ecology, University of Ulm, Ulm, Germany; 2 Smithsonian Tropical Research Institute, Balboa, Panama; 3 U.S. Geological Survey, Boulder, Colorado, United States of America; Chinese Academy of Forestry, CHINA

## Abstract

Figs (*Ficus* sp.) are often considered as keystone resources which strongly influence tropical forest ecosystems. We used long-term tree-census data to track the population dynamics of two abundant free-standing fig species, *Ficus insipida* and *F*. *yoponensis*, on Barro Colorado Island (BCI), a 15.6-km^2^ island in Lake Gatún, Panama. Vegetation cover on BCI consists of a mosaic of old growth (>400 years) and maturing (about 90–150 year old) secondary rainforest. Locations and conditions of fig trees have been mapped and monitored on BCI for more than 35 years (1973–2011), with a focus on the Lutz Catchment area (25 ha). The original distribution of the fig trees shortly after the construction of the Panama Canal was derived from an aerial photograph from 1927 and was compared with previous land use and forest status. The distribution of both fig species (~850 trees) is restricted to secondary forest. Of the original 119 trees observed in Lutz Catchment in 1973, >70% of *F*. *insipida* and >90% of *F*. *yoponensis* had died by 2011. Observations in other areas on BCI support the trend of declining free-standing figs. We interpret the decline of these figs on BCI as a natural process within a maturing tropical lowland forest. Senescence of the fig trees appears to have been accelerated by severe droughts such as the strong El Niño event in the year 1982/83. Because figs form such an important food resource for frugivores, this shift in resource availability is likely to have cascading effects on frugivore populations.

## Introduction

For many decades, tropical primary forests have been increasingly affected by forest conversion and degradation. In response, research into the regeneration and restoration of deforested areas and the conservation of associated endangered animal species is receiving increased attention [1–3]. This requires development of an in-depth understanding of the underlying complex relations among organisms in tropical ecosystems, and more information is needed with regard to species abundances, metapopulations, landscape dynamics, dispersal, and animal movement among different habitats, each of which may have a wide range of land-use histories [3].

Secondary forests are characterized by high turnover rates and distinct successional stages, with initial plant populations that consist of mostly short-lived, light-demanding and fast-growing pioneer species which are gradually replaced by a mature forest having mainly long-lived, slow-growing, and shade-tolerant species [1, 4]. Plant and animal species richness, but not necessarily species composition, may resemble those of mature forests within a few decades after land abandonment. The exact trajectory of change strongly depends, however, on land-use intensity, closeness to old-growth forests, and availability of seed dispersers [3, 5, 6].

Climate change, in particular the increasing frequency and severity of droughts associated with El Niño Southern Oscillation (ENSO) events, is another factor that may influence forest dynamics and may have a detrimental effect on ecosystems [7, 8]. In this context, long-term studies focusing on the history and development of secondary forests, or the investigation of changes in species composition both in primary and secondary forests provide insight into forest dynamics that may be crucial in predicting and potentially mitigating effects of short- and long-term changes in forest ecosystems and their associated fauna (e.g., [1, 3–5, 9–11]).

Some pioneer trees may be particularly important for forest regeneration or restoration because they attract and maintain large populations of frugivorous seed dispersers [6]. Figs (*Ficus* sp.) play such a role in many tropical forests because they are often pioneer species and frequently share characteristics associated with keystone species such as considerable abundance, year-round fruiting phenology with large fruit crops, and a high nutritional value [12–15]. Terborgh [13] even proposed that loss of figs could result in a collapse of forest ecosystems. Therefore, knowledge about the population dynamics of figs is crucial for a better understanding of fruit-frugivore dynamics in tropical forests, but may also provide a tool in support of forest management and conservation.

Barro Colorado Island (BCI) in Panama constitutes one of the most intensely studied tropical forests in the world, with research going back to the early 1920’s. It is at present covered by tall old-growth and secondary semideciduous rain forest at different successional states. Starting in the 1970’s, during long-term studies on frugivorous bats, researchers observed that the previously very abundant free-standing fig trees were declining, and complete inventories of all figs on the island were conducted. To verify the early observations, in 2006, we collected all available data about fig trees on the island and conducted additional fig mapping. Trees were mapped and tagged during the first census, and all subsequent studies always used these tag numbers, so that the fate of individual trees can be traced.

Here, we present data of this long-term monitoring (> 35 years) documenting the distribution and population dynamics of two species of free-standing figs, *Ficus insipida* Willd. and *F*. *yoponensis* Desv., with a focus on the Lutz Catchment, a 25-ha area where fig census data, starting in 1973, are available [14, 16, 17]. Although the number of trees has declined profoundly, both species once occurred in some areas on BCI at rather high densities with about 5 trees per ha [14, 16, 17]. Distribution and current status of the *Ficus* populations can be explained by historical land use patterns, and we discuss likely reasons for the drastic population decline and its implications for frugivores, in particular bats.

*Ficus insipida* has one of the highest measured photosynthesis rates in plants [18], which explains the very fast growth rates observed in this species [19, 20]. These figs can reach 30 cm dbh (diameter breast height) within 19–39 years, and may gain 90% of their maximum size (130 cm dbh) in about 52 years [19]. In Corcovado National Park, Costa Rica, trees of *F*. *insipida* with an average 85 cm dbh are quite abundant in 27 year-old secondary forests growing on former cattle pastures [21]. Although *Ficus insipida* colonizes natural levees along rivers and hillslopes of abandoned agricultural lands, it rarely colonizes blow downs, because the saplings require especially high-light conditions for successful establishment [19, 22]. Accordingly, *F*. *insipida* and likely also *F*. *yoponensis* are good indicators of past human land use. Because free-standing figs are both a pioneer species and a keystone resource for many animals, our observations may be relevant for forest restoration and conservation in degraded landscapes.

## Materials and methods

### Study area and land use history

**Fig 1:**
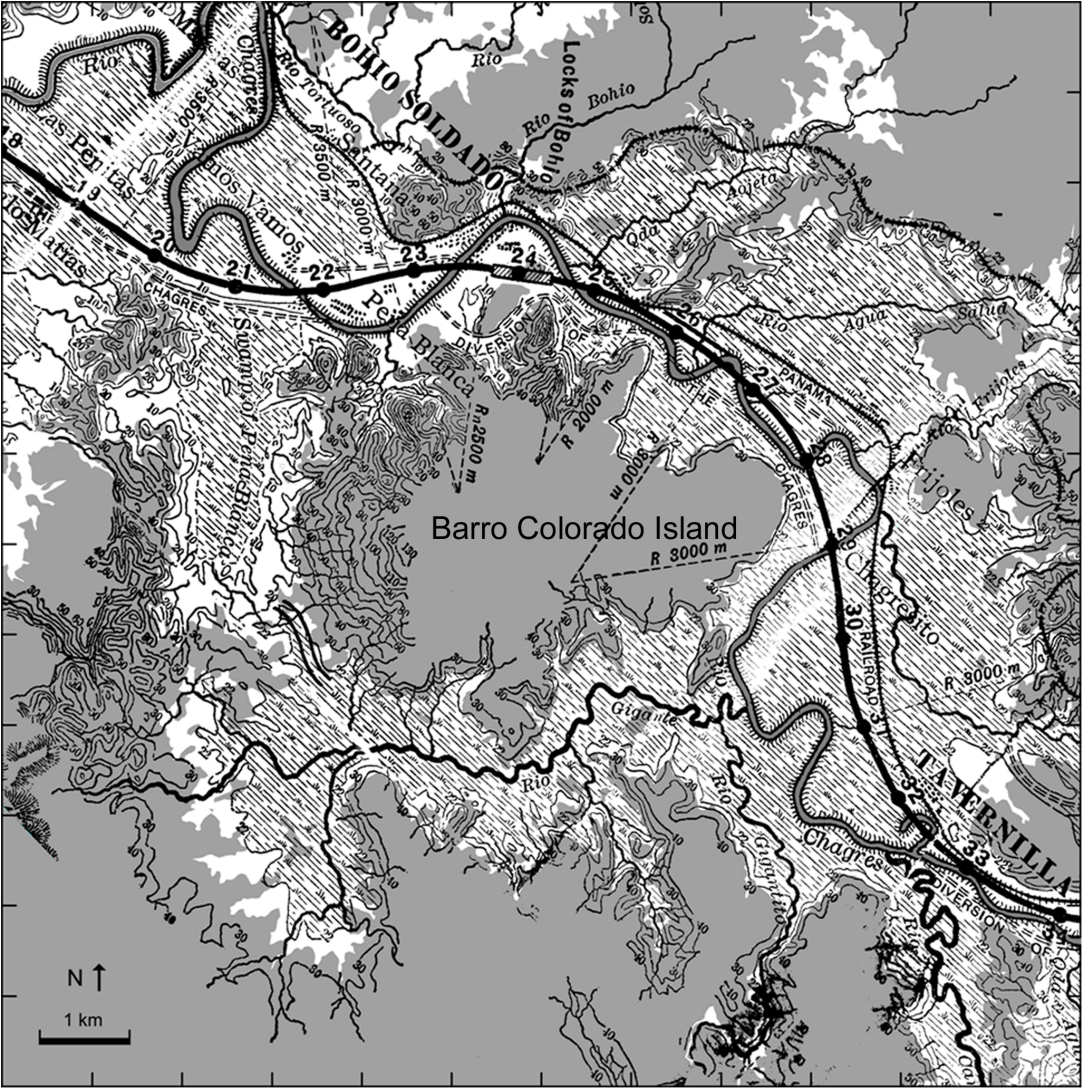
Map of the area around Barro Colorado Island before flooding (adapted from [38]). This map depicts the natural course of the Chagres River (flowing southeast to northwest) and positions of former settlements. Land areas remaining after the filling of artificial Lake Gatún in 1914 are shaded in gray. The remaining white background represents the modern lake. Also indicated are the railway to the north (the black dash-dotted line) and the planned course of the Panama Canal (thick solid black line). Topographic contours, from the original map, in meters above sea level.

Our study was conducted on the 15.6-km^2^ Barro Colorado Island (BCI) (9°09’ N, 79°51’ W), which was isolated in 1914 when the Chagres River was dammed to form Lake Gatún during the construction of the Panama Canal. Until 1914, BCI was a large hill at the lower Chagres River (Fig 1), surrounded by an extensive swampland fed by numerous small rivers through which the Chagres River meandered towards the Caribbean Sea from southeast to northwest.

BCI receives about 2,600 mm rain per year with a pronounced dry season (less than 100 mm per month) from mid December to mid April, during which only about 10% of the annual rainfall occurs [23]. The island is covered by a semideciduous rain forest of mixed ages that reflect a complex history of human settlement. Some findings indicate that BCI has been subject to agriculture for at least 7,000 years [24, 25], and most areas have been deforested at some point in the last 1,000 years [23]. Pot shards and putative grinding stones found on the island are clear signs of human occupation prior to European arrival ([22, 26]; R. Stallard, personal observation).

After the founding of Panama City in 1519, the Chagres River was a major route of migration and commerce. In 1858 the first ocean-to-ocean railroad was completed along the course of the lower Chagres River from the City of Colon to Panama City. As a result, much of the lower Chagres Valley was deforested and transformed into an agricultural landscape and new settlements were founded (see Fig 1).

In the late 1880’s, the French started excavations for a set of locks near Bohio Soldado [27], and were preparing to construct two major diversions of the Chagres alongside what is now BCI. This was presumably the time of greatest human population and impact along the north and east sides of BCI. The surrounding swamps and all the towns along the valley were flooded in 1914 by the creation of Lake Gatún.

In 1923, BCI became a natural reserve and a leading field station for tropical studies. Around that time the last agriculture land was abandoned [22, 28]. As a consequence of the pre-lake land use history, half of the island is currently covered with secondary forest mostly between 90 and 150 years old, but potentially up to 200 years old, and the south-western part is dominated by old-growth forest of least about 400–600 years old, and perhaps even over 2,000 years [22, 26].

### Classification of forest age

**Fig 2:**
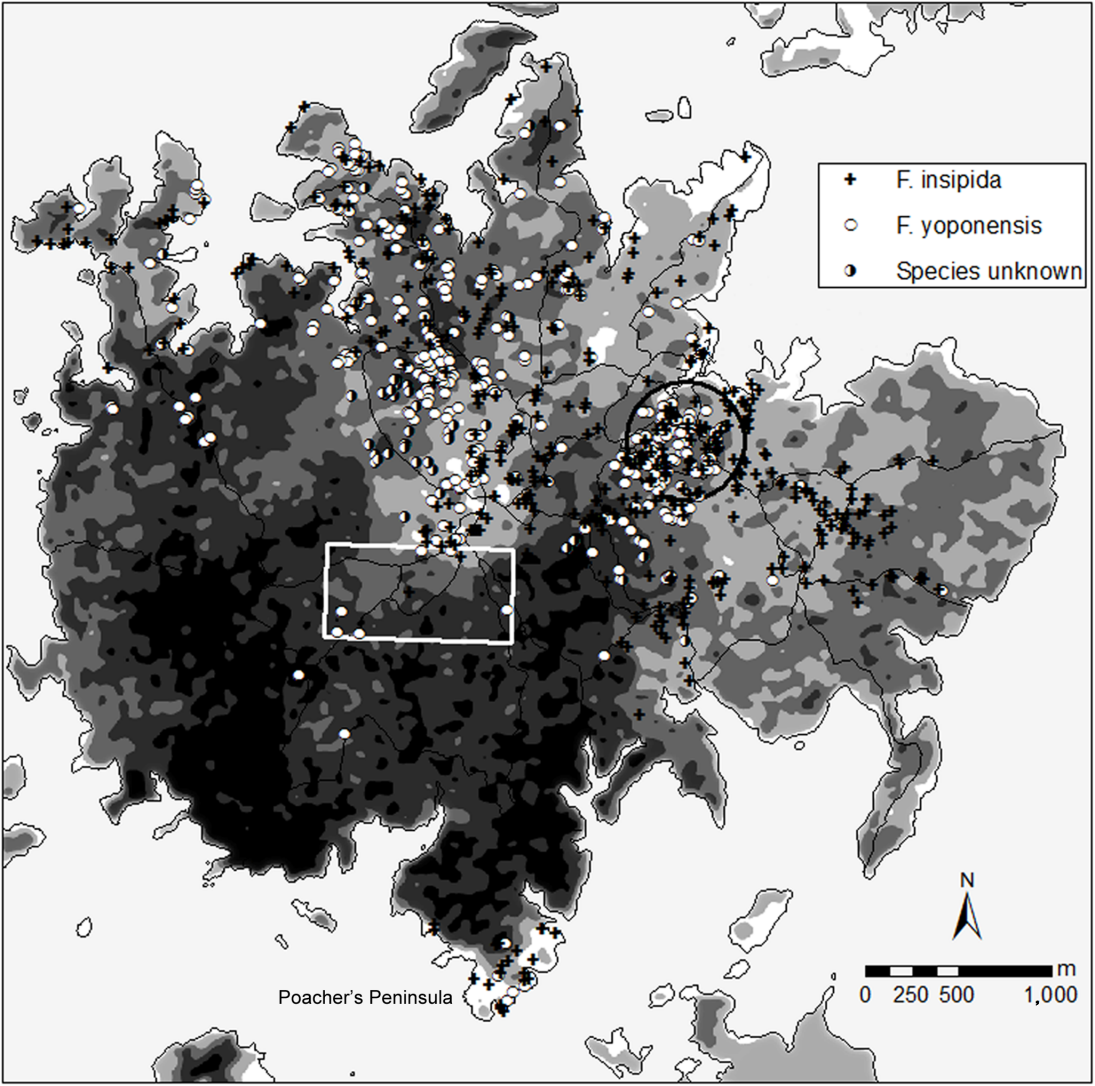
Distribution of *Ficus insipida* and *F*. *yoponensis* on Barro Colorado Island. Depicted is the relative age of the forest from young (white) to old (black) forest as determined from an aerial photo from 1927 (compiled by R. Stallard and D. Kinner 2002). Secondary forest is about 90–150 years old, primary forest at least 400–600 years. The black circle marks the position of the Lutz Catchment, the white rectangle indicates the 50-ha forest-dynamics plot.

The first reliable documentation of land cover on BCI dates from 1927. This documentation consists of a 1:10,000 topographic map [29] indicating forest, plantations, and non-forested land, and an aerial photo taken in early June 1927 [30]. Although there was no active rain gauge on BCI in 1927, rainfall data from [31] for the Canal Basin indicate that 1927 was an especially wet year, and that May 1927 was a particularly wet month. Accordingly, it is reasonable to assume that after more than a month of rain, the canopy density in the June 1927 photograph reflects full leaf coverage. The original oblique photo was digitally flattened, aligned with the current shoreline of BCI, and georeferenced for further use in a geographic information system (GIS). The rectified photograph was digitally smoothed and each pixel received a value between 0 (black, dense canopy) and 255 (white, cleared). The color values were divided into five classes (posterized): (1) 0–31, (2) 31–95, (3) 95–160, (4) 160–220, and (5) 220–255, depicting different forest ages. Isolated patches (<100 m^2^) were manually removed (Fig 2). Comparison with other BCI forest-age maps indicates that these categories correspond well to the forest disturbance categories distinguished on BCI [22, 30, 32]: old-growth (classes 1 and 2), tall-secondary (class 3), and low-secondary (classes 4 and 5) forest.

Early investigators mentioned that most of the secondary forest was probably about 50 years old when the reserve was founded [28, 32]. Based on the timeline and maps of the French activities, this would correspond to when the diversions were being constructed between the Chagres River and what is now BCI in the late 1880’s. Although the secondary forest now appears to be fully grown (2011), the height of the canopy is still increasing. A canopy tower of 42 m in height was constructed in the Lutz Catchment in 1972. By 2001, the canopy height had increased sufficiently to compromise the meteorological observations done on the original tower, and in October 2001, the tower height was increased to 48 meters [33].

### Data set

Adult fig trees were revisited and censused in the course of different studies over several decades to determine the fruiting phenology of figs in a selected area and to assess the fruit availability for fig-eating frugivores throughout the island. Although none of the fig censuses were performed to assess population dynamics of figs, condition of visited fig trees was generally noted and dead trees were always recorded. There are two data sets on figs available. In a 25-ha area called “Lutz Catchment”, all figs were censused frequently during 38 years (1973–2011). The second set encompasses all fig trees found during a census of the whole island between 1985 and 1987, which were then partially recensused twice in the subsequent 25 years. Although the second data set is incomplete, it reveals that the decline of free-standing figs is an island-wide phenomenon and not restricted to the Lutz Catchment, providing a broader basis for discussion about possible reasons of the decline.

The fig census was approved by the Smithsonian Tropical Research Institute (STRI) who administrates BCI and whose permission is required to perform any research activities on the island.

#### Lutz Catchment census

In 1973, a total of 142 fig trees encompassing six species were mapped in a 25-ha area called “Lutz Catchment” and tagged with individual numbers [16, 34]. Most of these trees (84%) belonged to the two free-standing fig species *Ficus insipida* (48 individuals) and *F*. *yoponensis* (71 individuals) (see also S1 Table). All individuals of these two free-standing fig species were mature and had a diameter of more than 50 cm. These trees were revisited at least once per census year (1973–1981, 1984, 1985, 1991, 1992, 1996, 2000, 2006–2011) in the course of different studies (e.g., [14, 16, 17]). In 2006 and 2007, we also searched systematically for new trees and saplings (> 50 cm in height) in the Lutz Catchment to determine whether there is any new recruitment of free-standing figs in this area. Between 2008 and 2010, all saplings were checked regularly and new saplings observed by chance were recorded.

Adult trees of *Ficus insipida* and *F*. *yoponensis* can be easily recognized from afar based on their smooth, grey trunks of up to 1.50 m in diameter with generally very conspicuous buttresses. Typically their distinctive leaves (dark, waxy green with bright yellow veins) can be found below the trees, so that even less striking trees are noticeable during systematic surveys. Saplings of both species have highly conspicuous, dark green leaves with striking yellow veins, about 20–40 cm in length and are thus easy to spot. We did not search for saplings smaller than 50 cm in height.

#### Island-wide census

From 1985 to 1989 C.O. Handley Jr. mapped *Ficus* trees across the whole island. Handley described the condition of each tree (stem size, trunk angle, presence of vines, crown condition, trunk condition, slope) and marked their positions on hand-drawn maps. All trees in the census had a trunk diameter of at least 50 cm and up to about 150 cm above buttresses. Most trees had a diameter of about 90–120 cm. These trees were revisited in 1991/92 by E.K.V. Kalko and marked with numbered aluminum tags as well as with two labeled strips of distinctive pink flagging, one attached to the tag nail, the other to a nearby small tree or vine. Unfortunately, paper copies of these census periods archived on BCI were not complete for all areas of BCI, and some maps only depicted positions of fig trees without species identification and/or tag number (see ‘Species unknown” in Fig 2). In total, data of about 140 *Ficus* trees tagged in 1991/92 (or later) were lost (most figs with the numbers 793–975). However, since Handley and others [14] noted that free-standing figs are scarce in the old-growth forest, we are confident that our general observations are still reliable despite missing data.

Most figs were then rechecked between 2006 and 2009 by L. Albrecht, and the accurate positions of all trees still present, or dead but identifiable, were geolocated using a high-resolution global positioning system (GPS) (Garmin 60CSx GPS, accuracy 5 m). Due to time constraints, about 280 of the original ~850 figs were not revisited in this last census. Positions of dead and decaying trees were often determined based on aluminum tags or the distinctive-pink flagging found on the ground or in the vegetation. Trees that were still alive were marked with new strips of labeled flagging on the tag and in the nearby vegetation. Trees without a tag (either new recruitment or where the original tag was overgrown) were marked with numerically consecutive aluminum tags following the census in 1991/92.

### Analysis

Information from paper copies of fig census data and maps archived on BCI were entered into Excel and mapped in the GIS-Program ArcMap 9.2 (ESRI Inc., USA), using the hand-drawn maps of C.O. Handley as a basis for the new census combined with the GPS locations determined between 2006 and 2009 to estimate positions for now-absent trees. We estimated the positions of completely vanished dead or decayed trees based on relative distances depicted in the hand-drawn maps compared to accurate positions of geolocated trees. Based on these data, we can determine the distribution of free-standing figs throughout the island. However, as the data set of the whole island is incomplete, statistical analysis of tree mortality could only be performed for the Lutz Catchment.

#### Lutz Catchment

Because of irregular census intervals and low sample size in the Lutz Catchment, we did not estimate mortality rates between census years and instead used the number of trees that were still alive for statistical analysis. In general, characterizations of time series should not make restrictive assumptions about the mathematical functions that are used to describe the data, because such assumptions can bias subsequent interpretation. Although we recognize that in a small population die-off is a series of steps, we assumed that data are smoothly varying. Spline functions are ideal computational tools for examining smoothly varying data, and we used relaxed cubic splines [35]. The relaxed cubic spline is a piecewise, continuous (connecting without gaps) set of cubic polynomials fit through the data, with one polynomial for each data segment; they are also piecewise continuous in their first derivative, used for rate calculations, and their second derivatives. With relaxed splines, the exact standard deviation is also specified. This standard deviation is chosen from other information, as we do below. The cubic spline regression gives the number of trees through time, n(*t*), where *t* is time in years. The time derivative, d n(*t*) d^–1^ *t*, gives the rate of die-off, and the mortality rate, λ(*t*), is (d n(*t*) d^–1^ *t*)•n(*t*)^–1^. The average annual mortality rate for a time interval was estimated from λ_*t*_ = 100•((ln(n(*t*_0_))—ln(n(*t*)))•*t*^-1^), where n(*t*_0_) is the number of trees alive at the beginning of the census period and n(*t*) the number of trees alive after t years.

Because the derivative of the tree count through time gives the rate of tree death, we must take care as to how the data used in the spline regression are prepared. The algorithm has a zero first derivative for the beginning and end of the data series [35]. To get a derivative for a starting point, the most useful technique is to double the length of the series and use the first point, (*t*_0_, *n*_0_), in the time series as a center of symmetry, whereby a synthetic series is calculated by reflection through this point. The synthetic points are labeled from –1 ≥–*i* ≥–n, such that *t*_–*i*_ = 2•*t*_0_ –*t*_*i*_ and *n*_–*i*_ = 2•*n*_0_ –*n*_*i*_. With this, the spline curve passes exactly through (*t*_0_, *n*_0_) and has a slope in keeping with the general trend of the data approaching that point. We also assume that at some time in the future, we chose 25 years, there will be no fig trees and therefore a zero tree count and zero derivative. The main source of errors is not so much missing trees, but the fact that the censuses were not done at precisely the same time within a census year. We chose the error to be half of the average number of tree deaths per year for each type of fig. Accordingly, the *a priori* standard deviation of *F*. *yoponensis* was ±0.86 and for *F*. *insipida* it was ±0.45.

## Results

### Lutz Catchment

**Fig 3:**
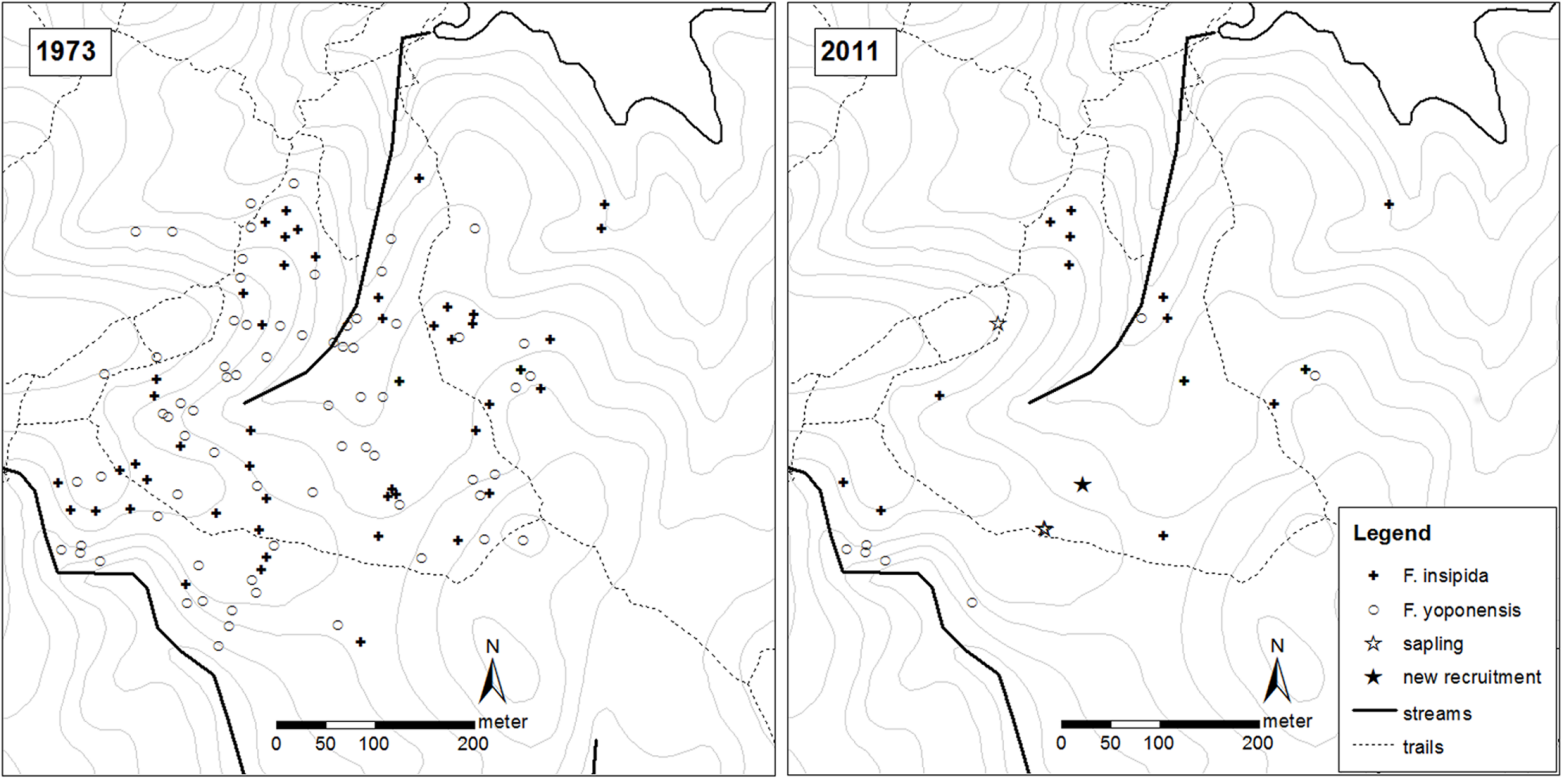
Results of the fig census in the Lutz Catchment. (a) Trees and saplings of *Ficus insipida* and *F*. *yoponensis* found alive in the Lutz Catchment in 1973 and (b) in 2011.

In 1973, 119 individuals of the two free-standing fig species were identified in the Lutz Catchment, 48 *F*. *insipida* and 71 *F*. *yoponensis* (Table 1, Fig 3A). By 2011, only 20–21 trees, 14 *F*. *insipida* and 6–7 *F*. *yoponensis*, were still alive (Table 1, Fig 3B). One *F*. *yoponensis* tree was not accessible due to impassable treefalls. In 2010, it was in a very poor condition, and in 2013, this tree was dead. Thus, between 1973 and 2011, more than 70% of *F*. *insipida* and 90% of *F*. *yoponensis* died. This results in an average annual mortality rate of 3.2% per year for *F*. *insipida* and 6.1% per year for *F*. *yoponensis*. Almost all of the remaining trees showed distinct signs of age, in that the trunk and/or the roots exhibited slight to severe signs of rot and frequently major limbs were broken off (for details, see S2 Table).

**Table 1:** Census data of each census year for *Ficus insipida* and *F*. *yoponensis* found in the Lutz Catchment. Given are the number of individuals of *Ficus insipida* and *F*. *yoponensis* still alive at each census and the percentage of individuals dead since 1973.

census year	*Ficus insipida*		*Ficus yoponensis*	
	# alive	% dead	# alive	% dead
1973	48	—	71	—
1974	47	2.1	70	1.4
1975	47	2.1	70	1.4
1976	47	2.1	70	1.4
1977	47	2.1	70	1.4
1978	47	2.1	69	2.8
1979	46	4.2	66	7.0
1980	46	4.2	65	8.5
1981	46	4.2	65	8.5
1984	40	16.7	55	22.5
1985	39	18.8	47	33.8
1991	36	25.0	33	53.5
1992	35	27.1	28	60.6
1996	31	35.4	18	74.6
2000	28	41.7	12	83.1
2006	20	58.3	8	88.7
2007	20	58.3	8	88.7
2008	19	60.4	8	88.7
2009	18	62.5	8	88.7
2010	17	64.6	8	88.7
2011	14	70.8	7–6 ^a^	90.1–91.5

^a^ One tree not accessible in 2011 due to treefalls was reported dead in 2013

In 2007, we found a comparatively small *F*. *yoponensis* (approximately 10–15 m tall, 45 cm dbh) that lacked conspicuous buttresses and had produced a small crown (about 10 m in diameter). This was the only new recruitment we found after an extensive search in Lutz Catchment. This tree is not included in the subsequent statistical analysis on mortality rates.

Three saplings of *F*. *insipida* were found in large gaps close to trails during the systematic search of the whole 25-ha plot. One sapling found in 2006 was still vigorous and fast growing in 2010, while two other saplings observed in 2007 had died within two years (the positions of all saplings are shown in Fig 3B). Throughout the census period of the Lutz Catchment from 2006–2011, we did not find any saplings in the forest under close canopy.

**Fig 4:**
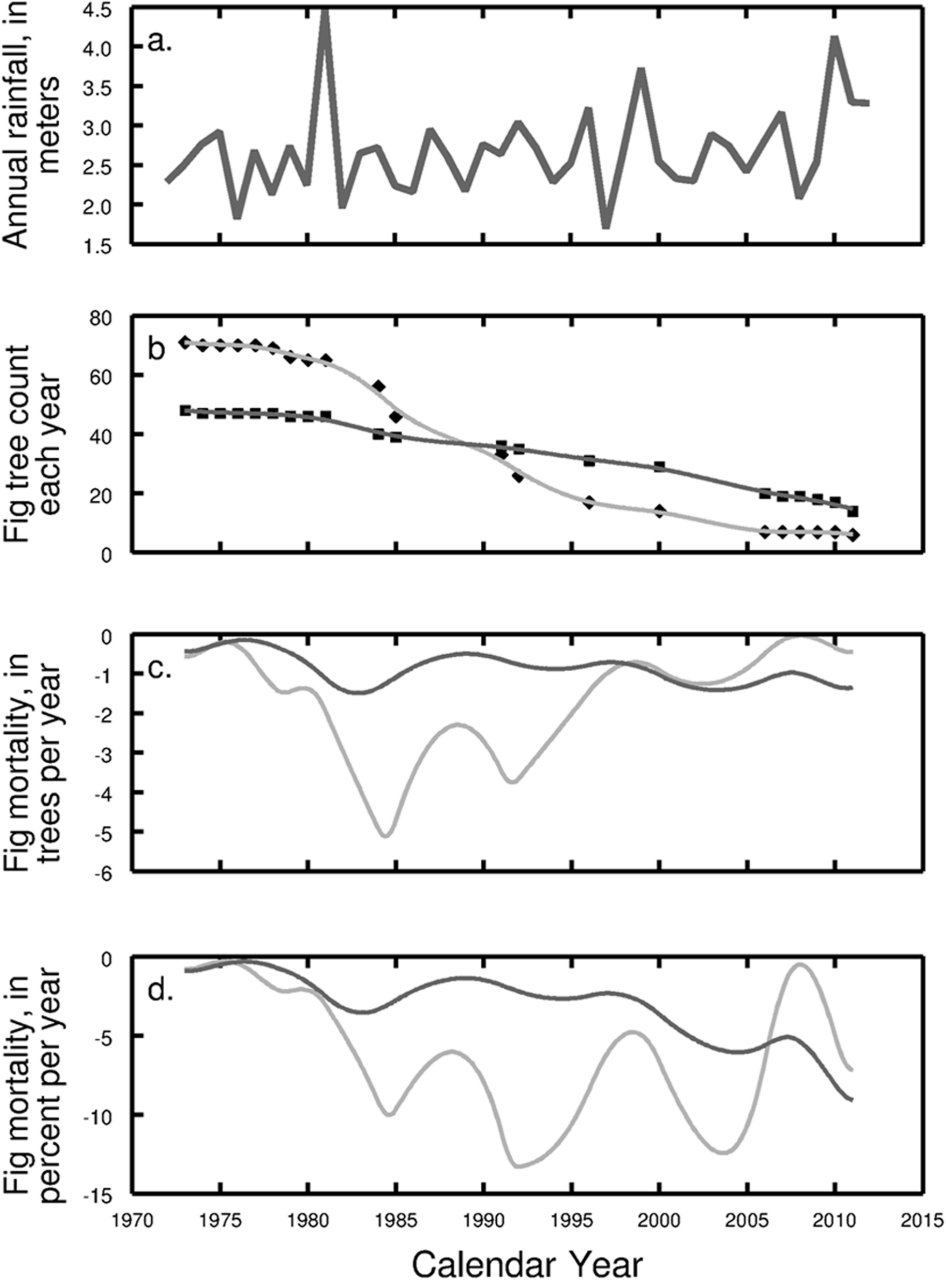
Mortality of fig trees in the Lutz Catchment. (a) Annual rainfall on BCI. Rainfall data are provided by the Environmental Studies Program (ESP). (b) Fig tree count per census year (light grey: *F*. *yoponensis*, dark grey: *F*. *insipida*). (c) Number of dead fig trees and (d) fig mortality in percent per year, estimated with relaxed cubic splines.

Mortality of trees varied strongly between census years, with periods of high and low mortality (Fig 4). Between 1973 and 1981 only eight of the free-standing figs had died, two *F*. *insipida* (Fig 4, dark grey line) and six *F*. *yoponensis* (light grey). Four of these trees were uprooted in a severe windstorm in 1979 [22]. High levels of mortality started in the early 1980’s, particularly of *F*. *yoponensis*. Seven *F*. *insipida* and 18 *F*. *yoponensis* perished within four years (1981–1985). For *F*. *yoponensis*, mortality remained high in the 1990’s, whereas the numbers of *F*. *insipida* started to decline more rapidly after year 2000. Interestingly, between 2006 and 2009, only two *F*. *insipida* and no *F*. *yoponensis* died, whereas between 2009 and 2011 four *F*. *insipida* and one *F*. *yoponensis* perished.

The relaxed-spline regression provides information about the rates and timing of the fig die-off. The mortality rate, λ(*t*), tends to be less for *F*. *insipida*, with greater (more negative) mortality around 1982 and a pronounced increase in mortality in recent years. *Ficus yoponensis* demonstrates several episodes of increased mortality, around 1985, 1992, 2004, and the end of the sampling. Similarly, mortality was increased in *F*. *insipida* around 1983, 1994, 2004, and the end of the sampling (Fig 4C and 4D).

#### Island-wide census

In total, almost 1,300 fig trees were mapped between 1985 and 1989 across the island. The census included 16 species: four free-standing fig species (subgenus *Pharmacosycea*) and 12 strangler fig species (subgenus *Urostigma*). The two most abundant species (>70% of all mapped trees) were *Ficus insipida* (>470 trees) and *F*. *yoponensis* (>365 trees) (see S2 Table). Most of the free-standing figs were growing in the secondary forest in the north-eastern part and the southernmost point of the island (Poacher’s Peninsula), with almost none in mature forest (Fig 2).

As of 1991/92, about 90 individuals of *F*. *insipida* (19%) and almost 100 individuals of *F*. *yoponensis* (27%) had died. By 2009, about 220 individuals of *F*. *insipida* (45%) and 180 *F*. *yoponensis* (48%) were definitively dead or were at least not found even after an extensive search during the most recent census (2006–2009). However, about 280 of the original ~850 free-standing fig trees were not revisited due to time constraints. Of those trees that were alive in 1992 and revisited in the census of 2006–2009, 48.4% of *F*. *insipida* and 37.8% of *F*. *yoponensis* were still alive (see S2 Table). Based on these data, we estimate that of the about 470 individuals of *F*. *insipida* and about 370 individuals of *F*. *yoponensis* found in the first census in 1985 on BCI, about 290 individuals (61%) of *F*. *insipida* and 270 individuals (70%) of *F*. *yoponensis* were dead by 2011. Thus, fewer than 300 of the about 850 free-standing figs from the year 1985 were likely still alive in 2011. Many trees visited between 2006 and 2009 were in a poor condition, with rotting roots and/or trunks and broken limbs. There was no area visited in which not most of the original free-standing figs were dead, and in some areas no living tree was found. We located very few free-standing fig trees without tag number, and only three trees were small enough (< 60 cm dbh), two *F*. *yoponensis* and one *F*. *insipida*, to count as new recruitments. While searching for figs, we also looked out for saplings. We observed nine saplings of *F*. *insipida* and one sapling of *F*. *yoponensis* of about 50 cm to 5 m in height.

## Discussion

Regeneration of deforested areas and its long-term implications on structure and composition of forests and the associated fauna have become important issues in tropical forest conservation worldwide (e.g. [3]). In this study on Barro Colorado Island, we demonstrate, using long-term census data sampled during different studies, that the transition from one successional state to another has the potential to transform the whole ecosystem by removing two keystone species, the free-standing figs *Ficus insipida* and *F*. *yoponensis*. Until a few decades ago, both figs were very abundant in the secondary forest of BCI, but their numbers have been declining rapidly since the early 1980’s. Our observations on distribution and population dynamics of these two fig species can be linked to land use history and the normal transition from a secondary to a more mature forest.

With its long record of human settlement, the forest of BCI is a mosaic of different stages of natural succession, and thus can provide insights into ecological processes of a maturing Neotropical secondary forest. Most of this secondary forest is more than 90 years old and is approaching old-growth forest in general appearance and structure, yet some long-lived pioneer trees, such as *F*. *insipida* and *F*. *yoponensis*, remain as indicators of a secondary forest.

### Population dynamics on BCI

In the Lutz Catchment, where a complete set of census data of 119 free-standing fig trees with frequent, though irregular census intervals are available, more than 70% of *Ficus insipida* and over 90% of *F*. *yoponensis* died between 1973 and 2011, while only one young *F*. *yoponensis* has successfully established (Fig 3). Accordingly, average annual mortality rate for the Lutz Catchment over 38 years (1973–2011) is very high, 3.2% per year for *F*. *insipida* and 6.1% per year for *F*. *yoponensis*. Thus, mortality rates for both species in Lutz Catchment are quite high and exceeded the normal long-term average of tree mortality rates in tropical forests of 0.5%-3.3% (e.g., [9, 10, 36, 37]). High mortality started in the early 1980’s after the 1982/83 ENSO event that caused a severe drought on BCI. Between 1981 and 1985, 18 of 65 *F*. *yoponensis* (27.7%) and seven of 46 *F*. *insipida* (15.2%) had died (see Table 1).

The decline of these two free-standing fig species is not a local phenomenon of the Lutz Catchment, but has been documented for the whole island. For all of BCI, of the ~850 free-standing fig trees that were identified around 1985–87, at least 400–600 individuals (45–72%) have perished, which results in an average annual mortality rate of approximately 2.0% for *F*. *insipida* and 1.3% for *F*. *yoponensis*, which is within the normal tree mortality rates in tropical forests. However, the island-wide census started in 1985, several years after a period of very high mortality observed in Lutz Catchment in the early 1980’s during and after the 1982/83 ENSO event, which particularly affected *F*. *yoponensis*. Because the drought affected the whole island [9], it is likely that many fig trees died before the census in 1985. Though repeat observations of many trees (28% of *F*. *insipida* and 39% of *F*. *yoponensis*) are lacking for the census in 2006–2009, in those areas revisited, many fig trees—sometimes all trees—were dead. Therefore, we conclude that the estimation that 61% of *F*. *insipida* and 70% of *F*. *yoponensis* have died since 1985 is justified. For *F*. *insipida*, the estimation is in the same range as observed in Lutz Catchment, where 64% of the trees died between 1985 and 2011. In Lutz Catchment, about 85% of *F*. *yoponensis* (40 of 47 trees) died since 1985, thus the estimation that 70% of *F*. *yoponensis* died on the whole island in the same time period may be conservative.

Overall, saplings were rare in the forest, and successful recruitment has been observed mainly along the lake shore or at the field-station lab clearing (L. Albrecht, personal observation). Altogether, very few young free-standing fig trees have been observed; thus there is virtually no replacement of dying fig trees. Free-standing figs need high-light condition for establishment, and presumably these trees rarely find suitable conditions to grow in older secondary or old-growth forest on BCI, and new recruitment is mainly restricted to forest edges. Our data indicate that *F*. *yoponensis* may be more successful in establishing in gaps, because several *F*. *yoponensis* trees, but no *F*. *insipida*, have been mapped in the old-growth forest areas (see Fig 2). Under the assumption that *F*. *yoponensis* exhibits similar growth rates as *F*. *insipida* [19], the only young tree (with 45 cm dbh) found in Lutz Catchment in 2007 might be about 20–40 years old.

With few exceptions, *F*. *insipida* and *F*. *yoponensis* were mostly in the secondary forest in the north-eastern part of BCI and on Poacher’s Peninsula in the south. Free-standing figs are pioneer trees [19, 22], and distribution of both species is associated with the young-forest side of edges between older-forest patches and younger forest (Fig 2), indicating that these figs were mainly growing along edges of pastures or agriculture land when the area was gradually abandoned. In the 1920’s, only a few trees in the deeper ravines in the secondary forest on BCI had stem sizes reaching 30 cm dbh [28]. Many free-standing figs in our study were located on top of hills or on flat areas, and some established on steep slopes. Mature fig trees have stem sizes of 60–150 cm dbh, thus most fig trees must have been very young in the 1920’s. Consequently, most individuals found on BCI most likely were established about 90–150 years ago, shortly after the land was cleared between the French attempt to build the Panama Canal in the 1880’s and the early 1920’s. By the early 1920’s, agriculture on BCI had been abandoned, and *Ficus* saplings had to establish before the then young secondary forest became too tall and dense. These pioneer free-standing fig trees remaining from that time are now old, exhibiting the typical signs of senescence such as rot on trunks and roots and broken main branches.

Before the creation of the Panama Canal, the Chagres River meandered through an alluvial floodplain [38] which probably was very similar in structure and forest composition to the floodplain area of the Rio Manu in Peru [4, 22]. In Cocha Cashu, lateral migration of meanders along the Rio Manu drives vegetation succession on recently deposited fluvial sediments, creating a chronosequence of different forest ages. In a transect of five 1-ha plots, from very young secondary forest close to the active river bank to an about 200 (±50) year-old mature forest, distinctive successional stages have been described [4, 19]. *Ficus insipida* and *Cedrela odorata* L. (Meliaceae) are saplings in the youngest hectare and dominate most of the secondary forest stages (hectares 2–4). In the oldest, about 200 year-old plot, *F*. *insipida* and *C*. *odorata* have almost been extirpated, and more shade-tolerant species dominate. Estimated mortality rates for *F*. *insipida* in the youngest to mid-successional stages in Cocha Cashu range from 1.4% to 5.6% per year [19]. Present-day populations of *F*. *insipida* and *F*. *yoponensis* on BCI correspond to the successional stages found in the fourth and fifth hectare in the Cocha Cashu plot, when long-lived pioneer trees such as free-standing figs are replaced by more shade-tolerant species.

It is likely that free-standing figs around BCI were naturally abundant in areas close to the meandering Chagres River before the Panama Canal was created, and some fig trees may also have been left as shade trees in the agricultural lands. These *Ficus* trees may have served as a seed source for the fast colonization of the abandoned agricultural land on BCI since the late 1880’s to the early 1920’s. Frugivorous bats, the main dispersers in early successional stages, usually carry fruits to a feeding roost up to 200 m away from the parent tree, creating a seed shadow of several hundred meters around the parent tree [6, 14]. The presence of such remnant fig trees in cleared areas may explain the often clumped distribution of figs on BCI (Fig 2).

Although the decline of free-standing figs observed on BCI is a normal process at this stage of succession, it does not necessarily happen smoothly. Our data indicate that mortality may have been increased by some episodic events such as drought (see. Fig 4). Periods of increased mortality coincide in both species, although *F*. *yoponensis* was affected more strongly, and mortality was highest after the severe drought caused by the very strong El Niño Southern Oscillation (ENSO) event in 1982/83, which also triggered enhanced mortality of many species in the 50-ha forest-dynamics plot in the middle of the island [9, 10, 39].

During the 1970’s fig mortality on BCI was low. Several trees died during a strong storm in 1979, which caused major localized blow downs [22]. High mortality rates started again in the early 1980’s (Fig 4) after the 1982/83 ENSO drought. Our data from the Lutz Catchment indicate that many fig trees died during or shortly after this event. Our calculations indicate that the peak in mortality for *F*. *insipida* was in 1983, at the end or shortly after the drought, while the peak for *F*. *yoponensis* was two years later. Individuals of *F*. *yoponensis* were most strongly affected, resulting in mortality of over 50% of the trees by 1991. The second and third die-offs of the figs in 1992/94 and 2004 do not seem to be linked to particular droughts, and neither species demonstrated immediate effects of the ENSO-related drought of 1997/98. The year 2008 was very dry on BCI, which may account for the increased mortality between 2009 and 2011 of both fig species after a period of low mortality (Table 1, Fig 4).

Generally, free-standing figs are not drought tolerant, because high photosynthetic rates correlate with high stomatal conductance and a rapid hydraulic water transport [18, 20]. Moreover, *F*. *insipida* is already close to maximum heat tolerance threshold during normal dry seasons [40], and increased temperatures during droughts may result in further stress. After the severe drought in 1982, many fig trees were probably weakened and thus more susceptible to wind damage, disease, rot, or beetle infestation, leading to death of individuals several years after this event [8, 41].

Although droughts probably accelerated the death of fig trees on BCI, we interpret the decline of free-standing *Ficus* trees on BCI as a natural part of the maturation of a secondary forest. The similarity to successional stages observed in Cocha Cashu and observations of Gautier-Hion and Michaloud [42] regarding figs in African rain forests corroborate this assumption. They noted that terrestrial figs in any rain forest evolving from second-growth stage to older stages will not be renewed, thus fig density and fruit production will strongly decrease. Consequently, these figs will cease being a stable food source for many frugivores when the forest reaches the late stages of maturation [42].

### Relevance for conservation

Free-standing figs are not only an integral part of many natural secondary forests, they may also serve as useful tools for forest management in fragmented landscapes. Trees of the genus *Ficus* have a very high impact on tropical forest ecosystems, particularly for the frugivore community (e.g., [13, 43, 44]). Overall, the abundance of figs is likely to be a main driver of abundance for many tropical frugivores (e.g., [44, 45]) which also has consequences on higher trophic levels. Although ripe crops appear to be widely scattered and unpredictable in time and space, the total fruit production per area is comparably high, as is the biomass of consumers [12, 14, 43, 46]. Because figs are fast-growing pioneer trees and very attractive for many seed-dispersing animals, planting of figs may aid in forest restoration in sites where natural regeneration is limited or needs to be accelerated.

Both *F*. *insipida* and *F*. *yoponensis* are very important food sources for frugivorous animals on BCI [14, 46, 47, 48]. They fulfill all requirements for keystone species: high abundance; year-round fruiting pattern, especially also during times of general fruit scarcity high fruit yield of up to 50,000 fruits per tree; and a high nutritional value (e.g., [14, 15, 17]). Thus, the disappearance of such a large number of figs will likely have an impact on the whole ecosystem on BCI.

There are many animal species feeding on figs on BCI, such as agoutis (*Dasyprocta punctata*), peccary (*Pecari tajacu*), kinkajous (*Potos flavus*), coati (*Nasua narica*), monkeys (e.g., *Alouatta palliata*, *Cebus capucinus*), frugivorous bats and frugivorous birds [14, 48]. Fig-eating bat species are among the most abundant bat species on BCI, likely due to the abundance of *Ficus* trees [14, 48, 49], with figs often comprising more than 70–80% of their diet [17, 50]. The most abundant bat species on BCI is the common fruit bat *Artibeus jamaicensis* (Leach, 1921) with an estimated population size of about 3,000 individuals on the island [14]. Although most fruit-eating bats consume quite a broad spectrum of fruits [14, 50], most of these fruits are only seasonally available, mainly during the rainy season (e.g., [14, 50]), and are usually eaten only when no figs are available [17]. Thus, in the future, if the population of *Ficus* trees becomes too small to produce reliable fruit crops during general fruit scarcity, the large populations of fig-eating bats on BCI will likely decrease due to food limitations [49, 50]. We assume that in 20–40 years, when the large free-standing *Ficus* trees have almost completely disappeared, the frugivore community on the island will have changed considerably, particularly the frugivorous bat guild.

There is no indication that other fig species can effectively replace *Ficus insipida* and *F*. *yoponensis* in terms of number or fruit availability. There are two other free-standing fig species known on BCI, *Ficus tonduzii* Standl. and *F*. *maxima* Mill. Both are scarce on the island with 45 and four individuals recorded, respectively, in the fig census of the whole island. Of seven *F*. *tonduzii* observed in 1973 in Lutz Catchment, six were dead by 1985, indicating that this species may have a shorter lifespan than *F*. *insipida* or *F*. *yoponensis*.

Strangler figs, which recruit on standing trees and thus also in mature forest, cannot entirely substitute for free-standing figs on BCI, because the densities of mature trees, number of fruits per crop as well as nutritional value are generally lower than in free-standing figs (e.g., [14, 15, 23, 51]). About 30% of the mapped trees in the census of the whole island were strangler figs, though the actual number may be underestimated. Most strangler figs are small, inconspicuous epiphytes and hemiepiphytes and may therefore be easily overlooked. In 2006–2010, some new, and mostly small, strangler figs were recorded, while several large strangler figs observed in earlier census periods were dead and gone. Overall, it is probable that all twelve strangler-fig species on BCI together may play an important role for wide-ranging, mobile frugivores such as large bats, but they will not have the overall quantitative importance of the two free-standing fig species *F*. *insipida* and *F*. *yoponensis*. No other non-fig species has the abundance and year-round fruit availability to compensate the loss of these figs for the fig-eating bat guild on BCI, and few species are fruiting during the dry season, which is a period of general fruit scarcity [14, 50].

On the other hand, figs, particularly strangler figs, are quite abundant on many of the small islands in Lake Gatun and on the nearby mainland (L. Albrecht, personal observation). Many frugivorous bats are able to commute over longer distances to find fruits [14, 17, 52, 53]. Thus, it may be possible that larger frugivorous bats, like *Artibeus jamaicensis*, will be able to maintain their population size on BCI, by expanding their foraging ranges when the availability of non-fig fruits on BCI itself is not adequate. Thus, it will be interesting to observe whether and how the ecosystem on BCI changes over the coming years following the disappearance of this keystone resource.

## Conclusions

Barro Colorado Island is an example of the regeneration of a Neotropical secondary rain forest over time following a long history of shifting agricultural land use. We found an obvious correlation between land-use history on BCI for the last 150 years and the distribution of the two abundant free-standing fig species *Ficus insipida* and *F*. *yoponensis*. Both fig species are a major food resource for many frugivores on BCI and are likely responsible for the high abundance of frugivorous bats on this island.

Now that the 90-to-150 year-old secondary forest on BCI is in a transitional stage between late secondary succession and a more mature forest, long-lived pioneer species, such as the abundant free-standing figs, are declining and shade-tolerant species are taking over. This transition, which affects the whole ecosystem, may be indicative for the future of many areas in the Neotropics that are recovering because of restoration associated with conservation programs. Generally, during secondary forest succession, tree composition changes drastically, often with a very high fruit resource density in middle to older successional stages due to the presence of free-standing figs and other pioneer fruit trees [19, 42]. Consequently, frugivore abundance will be highest in older secondary forests, and decrease together with the decline of figs when the forest matures (see also [21]). Overall, our observations on BCI may provide important information for long-term management and conservation of Neotropical forests and the associated fauna in times of increasing land-use pressure.

## Supporting information

S1 Table Fig census data from Lutz Catchment. Status of the free-standing figs (*Ficus insipida* and *F*. *yoponensis*) in Lutz Catchment (25 ha) at the respective census year. Indicated is also the source of data.
(XLS)

S2 Table Island-wide fig census data. Overview of the status of free-standing figs on the whole area of Barro Colorado Island during the respective census period.
(XLS)

S1 File Copyright of figures. (DOC)
