# Supplementary material for: Land use history and population dynamics of free-standing figs in a maturing forest
Source: PLoS One. 2017 May 24;12(5):e0177060. doi: 10.1371/journal.pone.0177060 (PMC5443483; doi:10.1371/journal.pone.0177060)
Supplement: S1 File — (DOC) [file pone.0177060.s003.doc]

**S1 file: Copyright of figures.**

None of the figures in this manuscript have been published in another journal or have been previously elsewhere copyrighted.

All map data used in the maps were prepared by entirely using US Government equipment, either at the USGS (most) or at the Smithsonian Tropical Research Institute (STRI; the initial scan of the vegetation map in Figure 2). As such, they are not copyrighted.

This is from the USGS FAQ web page on copyrights [<https://www2.usgs.gov/faq/categories/9761/3112>](https://www2.usgs.gov/faq/categories/9761/3112):

*"USGS products that are produced entirely within the agency are considered "public domain" materials; therefore, the public is free to use them in published material or in any other manner without obtaining consent or paying a fee. The USGS requests that the public adhere to some basic guidelines when using its materials. USGS materials should not be used or altered in such a way that they could potentially be perceived as being in a negative context. Whenever possible, the USGS asks that the use of USGS materials be cited somewhere in the text or manuscript. As a precaution, please make sure that the materials that you are crediting as having originated with the USGS were produced entirely by the USGS and not in cooperation with another source.*
